# Enhancing circuit stability under growth feedback with supplementary repressive regulation

**DOI:** 10.1093/nar/gkad1233

**Published:** 2024-01-02

**Authors:** Austin Stone, Sadikshya Rijal, Rong Zhang, Xiao-Jun Tian

**Affiliations:** School of Biological and Health Systems Engineering, Arizona State University, Tempe, AZ 85281, USA; School of Biological and Health Systems Engineering, Arizona State University, Tempe, AZ 85281, USA; School of Biological and Health Systems Engineering, Arizona State University, Tempe, AZ 85281, USA; School of Biological and Health Systems Engineering, Arizona State University, Tempe, AZ 85281, USA

## Abstract

The field of synthetic biology and biosystems engineering increasingly acknowledges the need for a holistic design approach that incorporates circuit-host interactions into the design process. Engineered circuits are not isolated entities but inherently entwined with the dynamic host environment. One such circuit-host interaction, ‘growth feedback’, results when modifications in host growth patterns influence the operation of gene circuits. The growth-mediated effects can range from growth-dependent elevation in protein/mRNA dilution rate to changes in resource reallocation within the cell, which can lead to complete functional collapse in complex circuits. To achieve robust circuit performance, synthetic biologists employ a variety of control mechanisms to stabilize and insulate circuit behavior against growth changes. Here we propose a simple strategy by incorporating one repressive edge in a growth-sensitive bistable circuit. Through both simulation and *in vitro* experimentation, we demonstrate how this additional repressive node stabilizes protein levels and increases the robustness of a bistable circuit in response to growth feedback. We propose the incorporation of repressive links in gene circuits as a control strategy for desensitizing gene circuits against growth fluctuations.

## Graphical Abstract

**Graphical Abstract:**
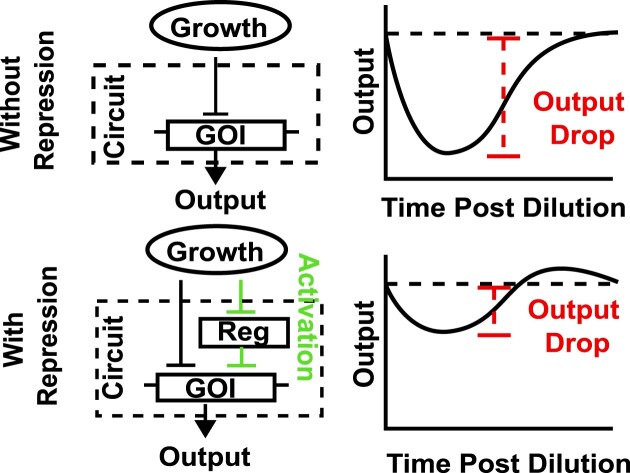

## Introduction

A commonality across engineering disciplines is the utilization of a general chassis or platform containing sets of commonly used structures upon which forward-engineered, application-specific protocols run. In synthetic biology, cells are often used as the platform on which genetic programs and circuitry run (1,2). However, as living systems are rarely static and exhibit abilities to grow, mutate, and respond to complex environmental signals, utilizing dynamic living entities as platforms for running forward-engineered programs poses a significant number of issues to predictability, robustness, and control of the desired application-specific constructs. Numerous forms of these so-called ‘circuit-host interactions’ have been identified and characterized, including resource competition, supercoiling mediated effects, growth feedback, etc. (3–14). The existence of these circuit-host interactions has led to an increasing emphasis being placed on the multi-scale modeling of these systems and recommendations for either holistic designs or the incorporation of component insulation techniques. These techniques fall under the umbrella of ‘host-aware design’ (5).

**Figure 1.**
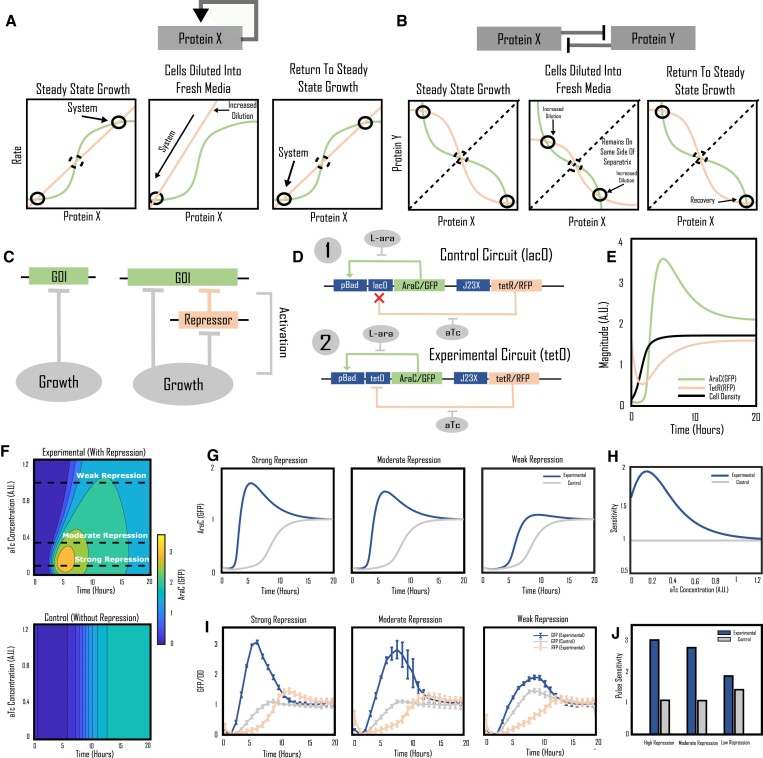
Repressive links add emergent pulse-like dynamics when paired with growth feedback. (**A**) Rate-balance plot of the self-activation switch. A system starting in the high state can be destabilized via transient growth, fall to the low state, and remain there even after growth returns to steady-state levels. The green and orange lines represent production and degradation rate curves respectively. (**B**) Nullcline analysis of the toggle switch. The symmetry of the circuit allows robust function during environments promoting global protein dilution. Green and orange curves represent Protein X and Protein Y nullclines respectively. (**C**) Schematic demonstrating the effect of a repressive link under growth conditions. Growth reduces the concentration of both the target gene of interest (GOI) and the repressor protein, which in turn simultaneously removes repression on the GOI resulting in a multiscale topology resembling an incoherent feed-forward (iFF) motif. (**D**) Schematic of the designed self-activation circuits. Circuits were composed of a self-activation module created by placing araC under the control of a pBad promoter with a GFP reporter, and a repressive module comprised of a TetR repressor tracked by an RFP reporter. Experimental and control circuits were differentiated by attaching a tetO or lacO sequence respectively to the pBad promoter. (**E**) Demonstration of the simulated response of the system in the off state (low GFP/AraC) to being diluted into new media with high L-ara concentration. The TetR/RFP concentration falls due to growth-enhanced dilution, while GFP/AraC concentrations rise due to exposure to high L-ara concentration. As the growth rate slows, TetR reaccumulates, resulting in GFP/AraC falling down from their peak to a lower steady-state value. (**F**) Simulation heatmaps of the response of the experimental and control circuit outputs over different aTc concentrations. Repression caused by TetR causes spiking behavior between 3–9 hours in the experimental circuit that is not seen in the control circuit; as aTc is increased (repressive strength of TetR removed), the peaking behavior disappears and the response of the experimental circuit approaches the control. (**G**) Comparison of experimental and control simulated growth responses for the aTc concentrations demarcated by the black dashed lines in Figure 1F. (**H**) Simulated pulse strength of the experimental and control circuits across different aTc concentrations. (**I**) Experimental data showing *in vivo* growth response of experimental and control circuits over different repressive strengths. RFP of the experimental circuit is also shown to demonstrate that the peak in GFP occurs during/shortly after the trough in repressor concentration, and GFP begins to fall as the repressor reaccumulates. Data normalized at their steady state. Bars show ± standard error (*n* = 4 biological replicates). (**J**) Pulse sensitivity of the experimental and control circuit responses shown in Figure 1I.

Growth feedback specifically is a phenomenon that universally affects living systems, and it is characterized by the effect changing host growth conditions have on circuit behavior (15–20). Changes in growth conditions can alter circuit behavior through a variety of mechanisms, including alterations to expression patterns, resource availability, resource distribution, and cell-to-cell variability (20–24). Particularly, increased growth rate has been demonstrated to universally increase protein dilution rates, as existing proteins are diluted across increasing population volume (19). For gene circuits containing multiple interactions between modules, this universal increase in dilution rate can introduce unintuitive behaviors into the system which are highly dependent on circuit topology. For example, both self-activation and toggle switch circuits can function as bistable switches, exhibiting hysteresis as they settle into distinct stable states depending on the initial conditions. However, our previous work demonstrated that the bistable self-activation circuit is particularly sensitive to this growth-mediated enhanced dilution while the bistable toggle switch can retain its memory under enhanced growth conditions (25). The reason bistable self-activation switches are prone to loss of function during growth can be visualized via a rate-balance plot as shown in Figure 1A. Here, intersections between protein production and degradation rates represent the steady states of the system where the rate of change of the protein concentration is zero. Under low growth conditions, a bistable self-activation system is represented by three intersections on its rate-balance plot, featuring two stable and one unstable steady state. Amplification in growth results in increased dilution rates, causing the slope of the degradation/dilution curve to significantly increase so that only one intersection point (steady state) can be found. If the growth rate is too high, the increase in the dilution curve could result in the disappearance of bistability and memory loss. On the other hand, a toggle switch with the same functionality but mediated via mutual repression was demonstrated to be significantly more robust to growth. The mutual repression in the toggle switch can buffer the system in a way that allows the system to retain its qualitative states. As shown in Figure 1B, when increased dilution is applied to both proteins in the system, the system does not lose any qualitative states. In addition, the qualitative states remain on the same side of the original separatrix (Figure 1B, dashed line). The separatrix demarcates the points within the phase diagram which are drawn towards distinct stable steady states. When growth is restored to a steady growth rate, the system recovers back to the original steady state.

Repressive links and negative feedback/feedforward control structures have been demonstrated to have a variety of stabilizing effects, including insulation from resource competition, noise attenuation, and leak control. It is hypothesized that it's for these reasons that negative control motifs such as negative autoregulation and negative feedback loops are among the most common regulatory motifs seen in nature (26–36). Ceroni *et al.* for example identified a stress/burden-responsive promoter in *E. coli* and placed a repressive moiety for synthetic gene production under the control of this burden-driven promoter, resulting in a negative feedback architecture with the ability to significantly stabilize synthetic protein production rate and host growth rate, albeit at the expense of significantly reduced synthetic protein production (37). Huang et al utilized a form of antithetic negative feedback by causing synthetic protein production to trigger the production of a repressive sRNA complimentary to the synthetic protein's mRNA to establish a form of quasi-integral control (38). Frei *et al.* demonstrated the viability of a more complex variety of negative feedback control (proportional-integral feedback control) by coexpressing the NES-L7Ae RNA-binding protein and an antisense RNA with the mRNA of the synthetic protein (39). Consequently, past studies have demonstrated the possibility of constructing diverse and complex control loop architectures for orthongonalizing genetic systems from their environment.

However, complex control loop architectures necessitate significant overhead both in terms of design and cellular resources. Furthermore, many stability design studies focus on stabilizing circuit behavior against fluctuating resource levels, input stochasticity, and resource competition between genes. Few investigate stability control in the context of transient growth dynamics and growth-dependent dilution of synthetic circuit moieties, a phenomenon that can significantly impact circuits that depend on the concentration of their mRNA/proteins to maintain memory and function. In this study we investigate the ability of the presence of simple repressive links without integration into a control loop architecture to attenuate the effects of host cell growth on circuit dynamics, utilizing a growth-sensitive bistable circuit as a model system. Throughout the study we utilize a bistable self-activation switch utilizing the pBad-AraC construct and simple GFP construct as our model circuits and study the effects of additional repressive interactions on the function of this circuit in the context of growth. We first demonstrate that the addition of repressive links can inherently add a pulse-like characteristic to the growth response of the gene expression in the circuit. This emergent pulse-like behavior can be utilized as a ‘drop rescue’ effect to attenuate how far the circuit activity falls during fast growth phase. Finally, we demonstrate that the output stabilization offered by this drop rescue effect can be utilized to increase the robustness of hysteresis behavior in bistable circuits against changing growth conditions.

## Materials and methods

### Simulation & modelling

Simulations performed in this study utilized ordinary differential equation (ODE) models to analyze the dynamics of cell density and protein concentration in the circuits. Simulations were run in MATLAB. The effects of growth feedback and cellular burden from resource consumption were taken into account in the model.

Modeling of the circuit: For modelling the circuit activity, only the outputs of AraC and TetR need to be modelled, as the production and degradation rates for the GFP and RFP reporters are assumed to be similar to the protein they are tracking. The production of these moieties includes a constant basal transcription term (${k}_{0,c}$ and ${k}_{0,t}$ for AraC and TetR respectively), and transcription-factor regulated term for the active production of AraC, which is activated by AraC (self-feedback, modelled by the function ${F}_A$) and repressed by TetR (modelled by the function ${F}_T$). Linear degradation is assumed with degradation constants ${d}_a$ and ${d}_t$:


(1) \begin{eqnarray*}\frac{{d\left[ {AraC} \right]}}{{dt}} &=& {\mathrm{\ }}\frac{{d\left[ {GFP} \right]}}{{dt}} \nonumber\\ &=& {k}_{0,a} + {k}_{1,a}{F}_A {F}_T - {d}_a\left[{AraC} \right]\end{eqnarray*}

(2) \begin{eqnarray*}\frac{{d\left[ {TetR} \right]}}{{dt}} = \frac{{d\left[ {RFP} \right]}}{{dt}} = {k}_{0,t} - {d}_t\left[{TetR} \right]\end{eqnarray*}

As the functions ${F}_A$and ${F}_T$ represent the activating binding of AraC to the pBad-tetO promoter and repressive binding of TetR to the pBad-tetO promoter respectively, ${F}_A$ takes the form of an activating Hill function with AraC as an input and with a binding term ${S}_A$ that represents the strength of binding between AraC and its cognate promoter. ${F}_T$ on the other hand, takes the form of a repressive Hill function with respect to TetR, whose strength is modified by the concentration of aTc. The forms of these two terms are explained in further detail in (25):


(3) \begin{eqnarray*}{F}_A\left( {\left[ {AraC} \right]} \right) = \frac{{{S}_a{{\left[ {AraC} \right]}}^2}}{{1 + {S}_a{{\left[ {AraC} \right]}}^2}}\end{eqnarray*}

(4) \begin{eqnarray*}{F}_T\left( {\left[ {TetR} \right],{\mathrm{\ }}aTc} \right) = \frac{1}{{1 + {{\left( {\lambda \frac{{\left[ {TetR} \right]}}{{{K}_t{{\left( {1{\mathrm{\ }} + {\mathrm{\ }}\frac{{aTc}}{{{K}_{aTc}}}\frac{{{K}_t}}{{\left[ {TetR} \right]}}} \right)}}^m}}} \right)}}^{{n}_t}}}\end{eqnarray*}

Here, ${K}_{aTc}$, ${K}_t$, $m$ and ${n}_t$ represent the aTc-TetR binding constant, TetR-promoter binding constant, aTc-TetR Hill coefficient, and TetR-promoter Hill coefficients respectively. For the control circuit, the function ${F}_T$ can be modelled as a constant by setting the hyperparameter $\lambda$ to zero.

The binding activity of AraC is increased dramatically by the presence of autoinducer L-ara. Consequently, the binding activity of AraC to promoter pBad can be modelled as an interpolation between its minimum binding activity (${C}_{min}$) and its maximum binding activity (${C}_{max}$), with the interpolation being mediated by an inducible Hill function of L-ara as described in (25):


(5) \begin{eqnarray*}{S}_a = C_a^{min} + \left( {C_a^{max} - C_a^{min}} \right){\mathrm{*}}\frac{{{{\left[ {Lara} \right]}}^n}}{{{{\left[ {Lara} \right]}}^n + K_a^n}}\end{eqnarray*}

*Modelling of growth and cellular burden*: The cell density $N$ of the system is modelled in accordance with simple logistic growth with the maximum growth rate ${k}_g$ and a carrying capacity of ${N}_0$. The effect of circuit resource consumption is taken into account by multiplying the rate given by this logistic form by a function ${F}_g$, which is a function of the concentration of output proteins and represents the effect of cellular burden on host growth:


(6) \begin{eqnarray*}\frac{{dN}}{{dt}} = {k}_g{\mathrm{*}}{F}_g{\mathrm{*}}\left( {1 - {\mathrm{\ }}\frac{N}{{{N}_0}}} \right)N\end{eqnarray*}

The constraints on function ${F}_g$ are 1) the value of ${F}_g$ must be unity when the circuit is not active (i.e. steady-state $[ {AraC} ]$ and $[ {TetR} ]$ are equal to zero), and 2) ${F}_g$ must be monotonically decreasing with respect to $[ {AraC} ]$ and $[ {TetR} ]$, approaching 0 as these terms increase. The simplest function ${F}_g$ that satisfies these conditions are:


(7) \begin{eqnarray*}{F}_g\left( {{P}_i} \right) = {\mathrm{\ }}\frac{1}{{1 + {\mathrm{\ }}\sum {\raise0.7ex\hbox{${{P}_i}$} \!\mathord{\left/ {\vphantom {{{P}_i} {{J}_i}}}\right.} \!\lower0.7ex\hbox{${{J}_i}$}}}}\end{eqnarray*}

where ${P}_i$ represent the proteins in the circuit (AraC/GFP and TetR/RFP), and the cellular burden factor J_i_ for AraC and TetR are assumed to be roughly equal.

*Modelling growth effect on protein dilutio* *n*: To model the last arm of the system, the effect of growth on the dilution rate of the circuit, the degradation rate of the proteins is increased by a term proportional to the cell growth rate:


(8) \begin{eqnarray*}d \to {d}_b + {\mathrm{\ }}\frac{1}{N}\frac{{dN}}{{dt}}\end{eqnarray*}

Here, ${d}_b$ represents intrinsic protein degradation rate while $\frac{1}{N}\frac{{dN}}{{dt}}$ represents dilution.

Parameters used in this model with their values and descriptions are shown in Supplementary Table S1.

### Strains, chemicals and media

DH10B and K-12 MG1655ΔlacIΔaraCBAD strains of *Escherichia coli* were utilized for gene circuit construction process and experimentation respectively. Cells were grown in 15 mL tubes at 37C on a shaker set to 220 RPM rotational speed in Luria-Bertani (LB) medium containing either 25 μg ml^−1^ chloramphenicol, 100 μg ml^−1^ ampicillin, or 50 μg ml^−1^ kanamycin. L-ara (Sigma-Aldrich) and aTc (Abcam) were dissolved into stock concentrations of 25% (volume-wise) and 1000 ng/ul respectively, then diluted with ddH_2_O into 1000× working solutions before being utilized for experimentation. Diluted media utilized to create the moderate growth condition during hysteresis experiments was created by mixing LB with M9 minimal media.

### Gene circuit construction

Circuits were constructed in either PSB1C3 (high copy number), PSB1A3 (high copy number), PSB3K3 (medium copy number), or PSB1K3 (medium copy number) backbones utilizing standard BioBricks parts from the iGEM registry via EcoRI, XbaI, SpeI and PstI restriction sites (restriction enzymes from ThermoFisher). RBS and terminators utilized for all operons were B0034 and B0015 respectively. Promoter and GOI sequences are listed in the Supplementary Information. The cloning protocol involved digestion with respective restriction enzymes (ThermoFisher), dephosphorylation with rSAP phosphatase (Sigma-Aldrich), followed by ligation with T4 DNA Ligase (New England BioLabs). Ligation products were transformed into DH10B strain of *E. coli* and screened for proper insert size via either colony PCR or EcoRI-PstI digestion. GelElute Gel Extraction Kit (Sigma-Aldrich), GenElute PCR Cleanup Kit (Sigma-Aldrich), and GenElute Plasmids Miniprep Kit (Sigma-Aldrich) were used for gel extraction, reaction cleanup, and plasmid extraction respectively. Sanger sequencing was used to confirm circuit sequences.

### Timeseries & hysteresis experiments

For time series and hysteresis experiments, cells were first transformed into the K-12 MG1655ΔlacIΔaraCBAD strain of *E. coli*, plated, and incubated overnight. Eight colonies were picked at random the next day for each circuit and inoculated into 5 ml culture tubes containing 300 µl of LB mixed with the respective antibiotics (25 μg/ml chloramphenicol, 100 μg/ml ampicillin, or 50 μg/ml kanamycin). Four of these tubes also had 2.6e-3% L-ara and 40 µL aTc to activate the cells into the ON state, while the other four did not, keeping the cells in the OFF state. Cells were grown for 8 h allowing the cells in the L-ara/aTc-present media to turn GFP-ON. After 8 h, cells were centrifuged at 2000g for 5 min and washed with and resuspended in 5 ml LB. 5 µl of cells were then loaded onto 96-well plates containing 200 µl systems with respective L-ara/aTc concentrations in each well. Plates were run on Synergy H1 Hybrid Reader (BioTek) for 10–20 h at either 28°C or 37°C, with the platform set to 807 CPM rotational speed. Cell density was measured via absorbance at 600 nm, while GFP and RFP were measured at 485/515 nm and 546/607 nm excitation/emission wavelengths respectively. Measurements were taken at either 15-minute or 30-minute time intervals. All experiments were run with four replicates.

The parameters for the high growth condition (37°C, 100% LB) for the hysteresis experiment were chosen to align with the reported optimal growth rate for *E. coli* (40). The temperature parameter for the low growth condition (28C, 5% LB) was chosen by setting our plate reader to the lowest temperature allowed by the incubator. The LB concentration was chosen to be the lowest concentration permitted without instigating the significant growth-dependent ribosomal dynamics as seen in ref (20). The growth rate constant of the low growth condition was measured to be around 0.2 h^−1^, approximately 70–75% lower than the growth constant at the high growth condition.

Concentrations of L-ara and aTc were picked by first beginning at value ranges around those utilized in ref (25) and then performing dose-response experiments to find a range of inducers that we thought was large and dense enough to show the full dynamic range of the promoters (i.e. the high end of the L-ara ranges chosen was picked to fall well into the plateau region of the dose-response curve). The working concentration ranges we utilized fell in the range of 0–2.6e-3% and 0–100 ng/ul for L-arabinose and aTc solutions respectively. We attempted to keep working concentrations of aTc to a minimum to prevent unwanted cytotoxic effects of aTc (range of 0–60 ng/ml for drop-rescue and hysteresis experiments). aTc concentrations above 600 ng/ml showed a marked drop in cellular signaling and survival rate.

### Flow cytometry

Flow data was collected on the Stratedigm S1000EON. 20000 events were recorded for each sample, with the absence of RFP utilized to gate out cells containing mutated or dysfunctional circuits. All samples were run with four replicates.

## Results

### Repressive links add pulse-like behavior to growth response

The schematics in Figure 1C show systems with and without repression under the influence of elevated growth conditions, and provides the rationale for our investigation. Without repressive regulation (left), the output of a gene circuit is repressed by an increased growth rate due to enhanced dilution during log phase growth. On the other hand, when the gene is placed under repressive regulation (right), a high growth condition not only increases the dilution rate of the target protein, but also increases the dilution rate of the repressor protein, removing repression on the target. In this fashion, when growth is included as a node in the overall block diagram of the system, placing a repressive link in the circuit converts the overall system into something resemblant of a multiscale incoherent feed-forward loop (iFFL), with growth simultaneously repressing the target gene directly and activating it indirectly through the repressor protein.

To study the effects of growth on these systems, two circuits were constructed as shown in Figure 1D (more details in Supplementary Figure S1 and Supplementary Tables S2 and S3) in *E. coli*. Both circuits contained a self-activating module constructed by placing gene araC under the control of a pBad promoter. As the pBad promoter already demonstrates all-or-none ultrasensitive expression, it makes for a strong candidate for constructing a model bistable self-activation switch (41,42). In the presence of the inducer L-ara, protein AraC can bind to and activate the pBad promoter resulting in a positive feedback loop. The activity of this node was reported by a green fluorescent protein (GFP) that was expressed alongside araC monocistronically. Both circuits also contained a repressive module comprised of a TetR repressor protein and a red fluorescent protein (RFP) reporter. The primary difference between the experimental and control circuits lies in the inclusion of either a tetO or lacO sequence in the pBad promoters, allowing the TetR protein to either bind (experimental) or not bind (control) to regulate the output of AraC and GFP. The repressive power of TetR can be tuned via the inducer aTc which can bind to TetR and deactivate its tetO binding capacity.

Due to the iFFL-like topology that circuits with repression demonstrate under elevated growth conditions, we hypothesized that initiating growth in a system containing a target gene repressed by repressor could result in a pulse-like behavior, as pulse behavior is a characteristic of iFFLs (43,44). To first explore the possible range of dynamics of the constructed circuits, we developed an ODE model of the system detailed further in the Methods section. Figure 1E demonstrates the simulated response of a cell population initialized in the OFF state (low GFP/AraC) with the repressive module active after dilution into fresh media with high L-ara concentration. The TetR/RFP concentration begins to fall shortly after the inoculation due to the elevated dilution rate, while GFP/AraC begins to rise as the cells are exposed to L-ara, eventually peaking. As the growth rate begins to decelerate, the TetR repressor begins to reaccumulate and the GFP/AraC concentration begins to fall from its peak down to a lower steady-state value. Figure 1F explores this behavior across a range of repressive strengths by testing the system over various aTc concentrations. Here, the control circuit remains unresponsive to aTc as the replacement of the tetO binding site with a lacO site prevents the TetR present in the cell from effectuating repression (Figure 1F, bottom panel). The behavior of the experimental circuit on the other hand changes drastically over the range of repressor strengths, showing an emergent pulse-like behavior for a low dose of aTc (Figure 1F, top panel). The time-series data at three different aTc concentrations (weak, moderate, and strong repression) are shown in Figure 1G. Notably, at strong repressive strength (low dose of aTc), the experimental circuit demonstrates a robust pulse-like behavior, and as repression is removed via increasing doses of aTc, the behavior of the experimental circuit approaches that of the control circuit. To compare the pulse-like characteristic across scenarios, we utilize a ‘pulse sensitivity’ characteristic, which is defined as the peak strength of the time-series curve after normalizing the steady state output level to unity. Figure 1H demonstrates the pulse sensitivities achieved by the experimental and control circuits across repressor strengths, with the experimental circuit demonstrating high sensitivity at low aTc concentrations and approaches that of the control as aTc concentration is increased.

To verify the predictions of our model, the experimental and control circuits shown in Figure 1D were inoculated into *E. coli* and grown in a medium with no L-ara to initialize the cells in the OFF state, and then inoculated into fresh media with a high L-ara concentration (2.6e-3% L-ara). Figure 1I (and Supplementary Figures S2 and S3 which display the unnormalized data) display time-series data for the GFP outputs of both the experimental and control circuits alongside the RFP output (a measure of the level of repressor in the cell) for the experimental circuit across three different repressor strengths (200, 400 and 600 ng/ml aTc). Notably, the experimental circuit displays elevated pulse behavior relative to the control with a high repressor strength, and gradually approaches the control circuit in behavior as repression is removed with aTc. Furthermore, this peak in GFP expression in the experimental circuit appears to happen during or shortly after RFP reaches its minimum, supporting a temporal link between the spike in GFP and the transient fall in repressor level. Pulse sensitivity data for these runs are shown in Figure 1J, consistent with the trend predicted by the mathematical model.

### Added activatory response demonstrates ‘drop-rescue’ effect and stabilizes output in bistable circuits

**Figure 2.**
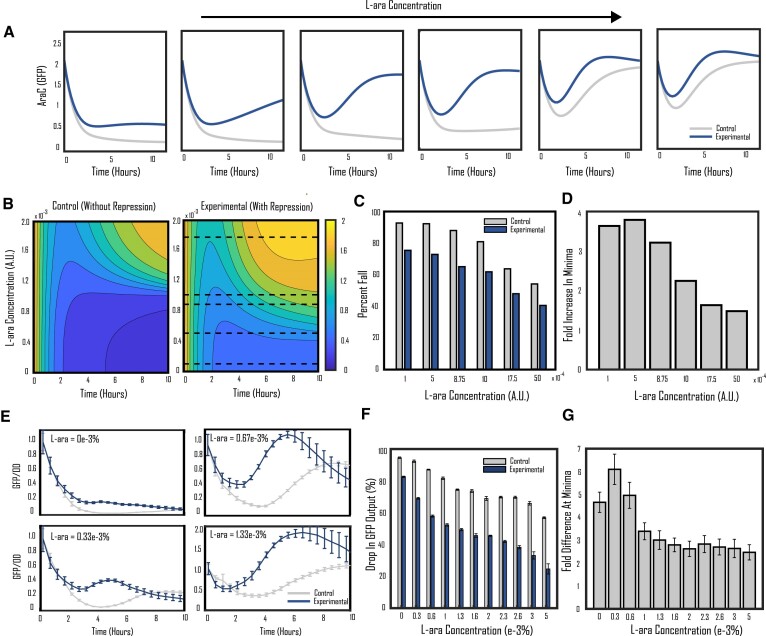
‘Drop-rescue’ effect of repressive links. (**A**) Simulated growth response of experimental and control circuits starting in the ON state (high GFP/AraC concentration) when diluted into fresh media with different L-ara concentrations. The output of the circuits falls due to increased growth rate, however the circuit with a repressive link is able to mitigate the drop in its output when compared to the control for all concentrations of L-ara. (**B**) Heatmaps of simulated growth response across various L-ara concentrations for both the experimental and control circuits. The experimental circuit is able to maintain higher output concentrations and lower output loss over most of the plane. (**C**) Simulated percent fall in output for experimental and control circuits within the first 10 hours of simulation at L-ara concentrations shown in the dashed lines in (B). (**D**) Fold increase in minima of the experimental circuit versus the control circuit at the L-ara concentrations shown at the dashed lines in (B). The Minima of the experimental circuit remain several folds higher than the control at all L-ara concentrations. (**E**) Experimental data showing *in vitro* response of the experimental and control circuits for four of eleven different L-ara concentrations tested. Experimental and control data normalized at their starting point. (**F**) Percent fall in output measured at the minimum during the first 10 hours of *in vitro* experimentation for all eleven L-ara concentrations tested. (**G**) Fold increase in the minimum of the experimental circuit compared to the control circuit *in vitro* data during the first 10 hours. The experimental circuit maintains minima several fold higher than that of the control. All error bars in (E–G) show ± standard error (*n* = 4, biological replicates).

Given the ability of a repressive link to add an activating component to a gene's growth response, we hypothesized that this added behavior could be used to buffer the fall in gene expression output during growth. Figure 2A shows our ODE model's predicted response of the circuits with and without repression starting in the ON state (high GFP/AraC) and diluted into fresh media containing different concentrations of L-ara. The circuit output falls in the period following the introduction to new media due to the increased dilution rate. At low L-ara concentrations, the self-activation switch cannot sustain itself during this period of output loss and quickly falls to the OFF state. As the L-ara concentration rises, the initial drop in output caused by growth becomes less significant and the self-activation circuits have an elevated capacity to recover back to the ON state. The experimental system not only retains its output more robustly when compared to the control circuit across all trials, but the added repressive link in the experimental circuit demonstrates the ability to mitigate the initial output drop caused by high growth. Figure 2B shows the simulated responses of the experimental and control systems across a wide range of L-ara concentrations, with the experimental circuit retaining elevated output and achieving higher minima for the entirety of the plane when compared to the control circuit.

To visualize the difference in behavior between the two circuits, we utilized two metrics to gauge the performance of the experimental relative to the control: (i) output loss measured by comparing the minima of the time-series responses to the starting output (Figure 2C), and (ii) the fold difference between the output minima achieved by the experimental versus the control circuits (Figure 2D), both metrics being assessed within the first 10 hours of simulation. Calculating these metrics at the L-ara concentrations shown by the black dashed lines in Figure 2B, the experimental circuit shows both significantly attenuated output loss and minima several-fold higher when compared to the control for all L-ara concentrations. The increase in fold minima of the experimental circuit peaks for mid-range L-ara concentrations as the ultrasensitivity of the pBad promoter allows the small advantage the experimental circuit has to be amplified in this region, with a drop in the fold increase in minima afterward as the control circuit catches up.

To verify the trends predicted by the model simulations, we first activated our experimental and control circuits in media with a high concentration of L-ara to the ON steady state (high GFP/AraC) before inoculating them into fresh media across eleven different L-ara concentrations. Figure 2E (unnormalized data in Supplementary Figure S4) shows the time-series data for cells diluted into fresh media containing four of the eleven L-ara concentrations tested: 0%, 0.33e-3%, 0.67e-3%, and 1.33e-3%. At 0% L-ara, the circuit does not have enough inducer to sustain itself in the ON state through the growth period and quickly turns off for both the experimental and control circuits, similar to the simulation in Figure 2A. As the L-ara concentration increases, a temporary drop in output was found for both the experimental and control circuits during the growth phase but it rebounded quickly to the fully ON state. The experimental circuit for all these trials demonstrates a heightened response when compared to the control. Figure 2F and G display the percentage fall in output and fold difference in minima between the experimental and control circuits for the first 10h of experimentation for all eleven concentrations of L-ara. The experimental circuit is able to mitigate its output drop better than the control circuit for all L-ara concentrations and attain minima several-fold higher than the control levelling out at around 2.5× the control minima for high L-ara concentrations.

To validate the generality of the ‘drop-rescue’ effect, we constructed two simple circuits without feedback loops (Supplementary Figure S5). In these circuits, we only have GFP as a general gene of interest under the expression of a constitutive promoter combined with either a TetO or LacO regulatory sequence, which can be substituted by more intricate genetic circuits. In the experimental circuit, the controller node TetR binds to the GFP promoter to inhibit its transcription, while in the control circuit, TetR does not interact with the GFP promoter. aTc concentrations that result in matching activity between the experimental and control circuits were found (Supplementary Figure S6), which allowed the following experiments to be run in a comparable setting. To test the difference in growth response between these simple experimental and control circuits, we first set the circuits into a steady state with 8 hours of induction of ATc and then diluted them into fresh media to measure the GFP dynamics. As shown in Supplementary Figure S7A, the GFP expression level shows a drop in both circuits before rebounding back to the steady state, but the decline is notably less pronounced in the experimental circuit when compared to the control circuit. This behavior was confirmed by two other pairs of experimental/control circuits utilizing different promoter pairs (Supplementary Figure S7B and C). This demonstrates that the ‘drop-rescue’ effect is a general phenomenon achievable through our control strategy.

### Drop-rescue effect can enhance hysteresis robustness against growth in bistable circuits

Hysteresis collapse occurs when the bistable system is unable to maintain protein levels above the separatrix due to enhanced dilution during log phase growth. As the incorporation of a repressive link demonstrates some ability to buffer against the drop in output and protect the circuit output from falling below the separatrix, we hypothesized that the same mechanism could be utilized to increase the robustness of hysteresis during growth and help prevent hysteresis collapse.

**Figure 3.**
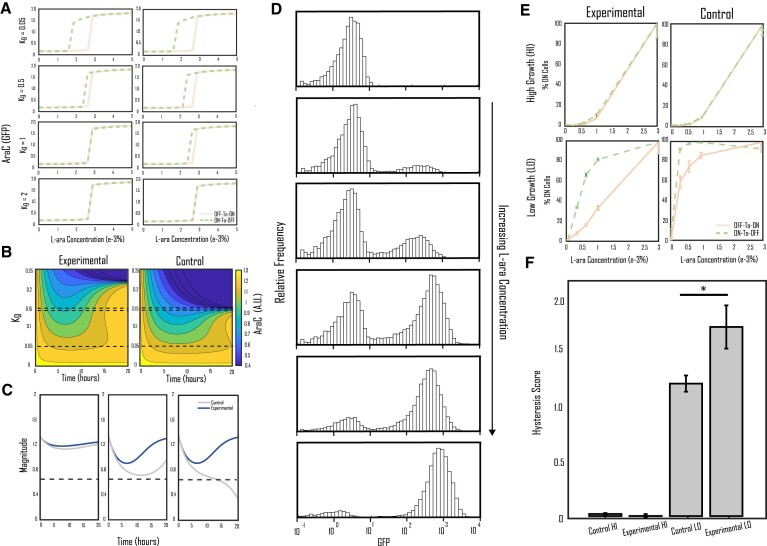
Evidence that drop-rescue effect can stabilize hysteresis robustness against growth in bistable circuits. (**A**) Simulation results showing the effect of increasing growth rate on circuit bistability on both the experimental and control circuit architectures. Both circuits show a reduction in the robustness of hysteresis as the growth rate increases, however the reduction in hysteresis robustness seems to fall slower in the experimental circuit versus the control. (**B**) Heatmaps showing simulated time-series data for cells starting in the ON steady state diluted into fresh media over increasing growth rates. (**C**) Comparison of time-series data of experimental and control circuits at the growth rates denoted by the black dashed lines in (B), with respect to the circuit's separatrix. (**D**) In vivo flow cytometry data of the self-activation module over increasing L-ara concentration. The self-activation module displays strong bimodality in output, as the frequencies around the unstable steady state remain low for all L-ara concentrations. (**E**) Hysteresis curves for *in vivo* experimentation at moderate (28°C, 5% LB) and high (37°C, 100% LB) growth rates. No hysteresis is present at a high growth rate, and hysteresis begins to emerge as the growth rate is reduced. Graphs are normalized by setting their highest value to 100% ON. (**F**) Quantification of hysteresis scores for the four hysteresis curves in (**E**). Hysteresis scores for the high growth conditions are practically zero, while the hysteresis scores for the low growth condition are significantly higher than zero, signifying the emergence of bistability. The score for the experimental circuit at this condition is higher than that of the control (*P* = 0.029). All bars in (E) and (F) show standard error (*n* = 4, biological replicates).

Figure 3A shows the model's prediction of this behavior on the dose-response diagrams of the experimental and control circuits with ON or OFF initial conditions. At a low growth rate (${k}_g$), both experimental and control circuits demonstrate large bistable region size. As the growth rate increases, the bistable regions of both circuits begin to shrink, however, the shrinkage for the experimental circuit remains much slower than for the control circuit. At ${k}_g$ = 1, the hysteresis of the control circuit has completely collapsed while the experimental circuit retains some of its bistable region, and at ${k}_g$ = 2 the growth rate is high enough that neither of the circuits retains memory. Consequently, our model predicts that the incorporation of a repressive link could increase the insulation of bistable circuits against growth feedback.

Figure 3B demonstrates model prediction of how these alterations in the bistable curves stem from the transient dynamics of the system. For these simulations, the simulated systems were initialized to the ON steady state and then inoculated into fresh media. When the growth rate is set to zero (${k}_g$ = 0), there is no change to the circuit output as the circuit remains at its steady state. As the growth rate increases, a transient dip in output begins to form, although both circuits are able to recover and are attracted to the ON steady state they started in. However, above a certain growth rate, the production rate of the system can no longer keep up with the growth-dependent increase in dilution and the output of the system falls precipitously to the OFF state (dark blue). Notably, this threshold occurs sooner for the control circuit (${k}_g$= 0.15) than for the experimental circuit (${k}_g$ = 0.23). Figure 3C shows the time-series comparison between experimental and control output in relation to the circuit's separatrix for the growth rates shown by the black dashed lines in Figure 3B. At the moderate growth rate level (${k}_g$= 0.13), the output of the control circuit dips dangerously close to the separatrix while the drop-rescue effect active in the experimental circuit keeps it well away from the separatrix. At a slightly higher growth rate (${k}_g$ = 0.16), the drop in output of the control circuit is just enough to push the system over the separatrix, resulting in the control circuit switching to the OFF state while the experimental circuit retains its memory.

To test the predictions made by the model, cells containing either the control or experimental circuit were assessed for bimodality and hysteresis via flow cytometry measurements. Figure 3D shows cytometry data for the reporter GFP over various L-ara concentrations (0%, 0.67e-3%, 1e-3%, 1.33e-3%, 1.67e-3%, 2.33e-3% L-ara), demonstrating the robust bimodal distribution necessary for hysteresis. The cells increasingly switch from the OFF to the ON state with increasing L-ara concentration, with intermediary states surrounding the unstable steady state between the ON and OFF regions displaying low probability density for all concentrations. Bistability was quantified by growing cells containing either control or experimental circuits to either the OFF (low L-ara) or ON (high L-ara) steady states, then diluting them into fresh media with various L-ara concentrations and measuring the percent ON cells (described in further detail in the Methods section). As shown in Figure 3E, this process was performed under two different growth conditions: a moderate growth condition (28°C, 5% LB) and a high growth condition (37°C, 100%LB). The growth rate constant of the low growth condition is 0.2 hour^−1^, approximately 70–75% lower than that at the high growth condition (Supplementary Figure S8). Figure 3E shows the data after the points with the highest percentage of ON cells were normalized to 100. It can be easily seen that under the high growth condition, neither circuit displays hysteresis behavior as the growth rate is far too high to preserve information with this topology. On the other hand, at the moderate growth condition, hysteresis behavior begins to emerge as cells are able to retain their memory past the log growth phase.

The precise range of the bistable region for the utilized self-activation switch is difficult to directly compare due to noise and other factors. Consequently, to score the hysteresis curves on how strongly they resemble hysteresis behavior, we find the area between the off-to-on and on-to-off curves of the diagram generated from the normalized flow cytometry data. Figure 3F shows the calculated hysteresis scores for the four scenarios; hysteresis scores for both high-growth state scenarios hover close to zero, while hysteresis scores for moderate growth rate are positive with the experimental circuit trials performing better on average than the control circuit trials. These findings support the conclusions drawn from the simulation results.

## Discussion

Systems biology is rife with difficult and unintuitive interaction patterns, making it both a difficult and exciting field of investigation. As growth feedback is a prime example of the importance of multi-scale interactions in systems biology, the phenomenon itself can be studied from two distinct levels. On the population level, both the total cell mass and the system volume increase as cells grow. This growth-associated expansion results in the dilution of the circuit protein concentration. At the single-cell level, however, the phenomenon of growth-dependent dilution manifests as follows: before each cell division, the individual cellular volume enlarges, leading to a dilution of the protein concentration within the cell. This diluted concentration is subsequently inherited by both daughter cells. Although deterministic modelling was utilized as a rigorous predictive aid throughout our study, it is important to acknowledge that the growth-dependent dilution process is inherently stochastic at the single-cell level. Two key sources of variability contribute to this stochasticity: (i) variability in division times: different cells may exhibit significant variations in the timing of cell division, and (ii) asymmetric partitioning: the distribution of protein and mRNA copy numbers between daughter cells during cell division may not occur in a strictly proportional manner (21,45–47). At the population level, much of this inherent stochasticity averages out, resulting in the emergence of a population-level dilution rate closely linked to the overall growth rate.

Living systems pose unique challenges to engineers, as cells provide platforms for gene program execution that are not static but rather growing and constantly in flux. The results of this study further underscore the importance of considering circuit-host interactions in the design of synthetic biological systems, as the effects of changing growth conditions on circuit function reduce the predictability of circuit behavior and require considerations of circuit-host design. In a previous study, we demonstrated that the negative effects of growth feedback are in some capacity dependent on the circuit topology, wherein bistable self-activation circuits were shown to lose their hysteresis quickly while bistable toggle circuits display bistable behavior that is more robust to growth (25). This work also implies that incorporating an additional positive feedback loop would not be a good strategy to mitigate these dilution effects. Given previous evidence on the potential stabilizing effects of repression, in this study, we demonstrate that repressive links in network motifs inherently provide buffering capacity against growth feedback and stabilize circuit output against growth-dependent changes in protein dilution rate. We utilize both mechanistic mathematical modelling and experimentation to demonstrate that this buffering capacity can be utilized to reduce the growth-dependent drop in circuit protein output, and that this ‘drop-rescue’ effect can be used to stabilize circuits that are sensitive to drops in output, such as bistable self-activation switches. Given the simplicity and inherent insulation capacity of repressive links, the addition of auxiliary repressive links can be utilized as a design principle for combatting growth-feedback phenomena.

## Supplementary Material

gkad1233_Supplemental_File

## Data Availability

The data underlying this article are available in the article and in its online supplementary material.
