# Supplementary material for: Enhancing circuit stability under growth feedback with supplementary repressive regulation
Source: Nucleic Acids Res. 2024 Jan 2;52(3):1512–21. doi: 10.1093/nar/gkad1233 (PMC10853785; doi:10.1093/nar/gkad1233)
Supplement: gkad1233_Supplemental_File [file gkad1233_supplemental_file.pdf]

Supplementary Material

Enhancing Circuit Stability Under Growth Feedback with Supplementary Repressive Regulation

Austin Stone[1], Sadikshya Rijal[1], Rong Zhang[1], Xiao-Jun Tian[1*]

[1]School of Biological and Health Systems, Engineering, Arizona State University, Tempe, Arizona 85281, United States.

* To whom correspondence should be addressed. Email: xiaojun.tian@asu.edu

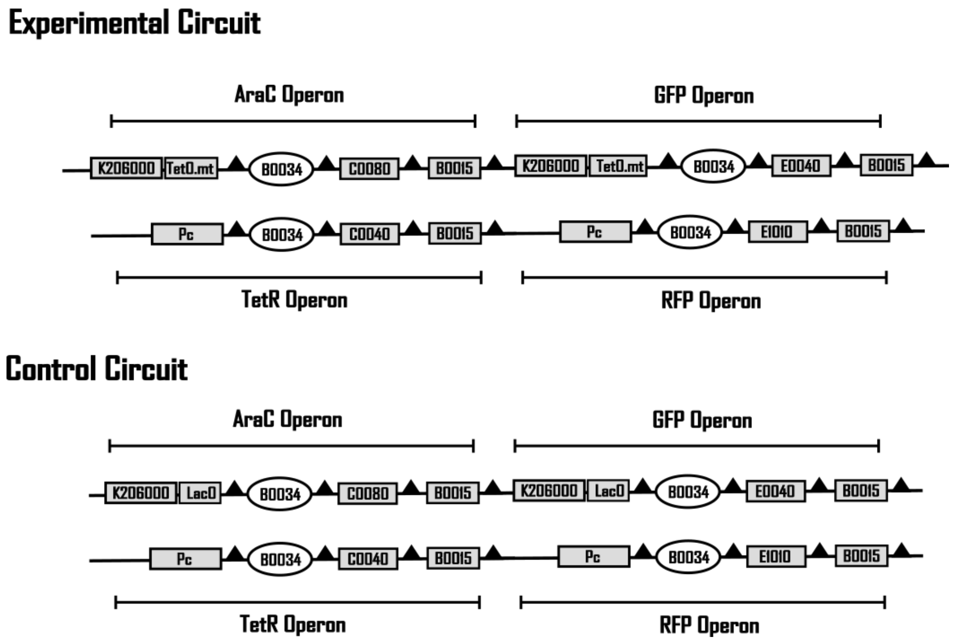

Figure S1. Detailed design of the experimental and control self-activation circuits. The sequence information for circuit design can be found in Table S2-3.

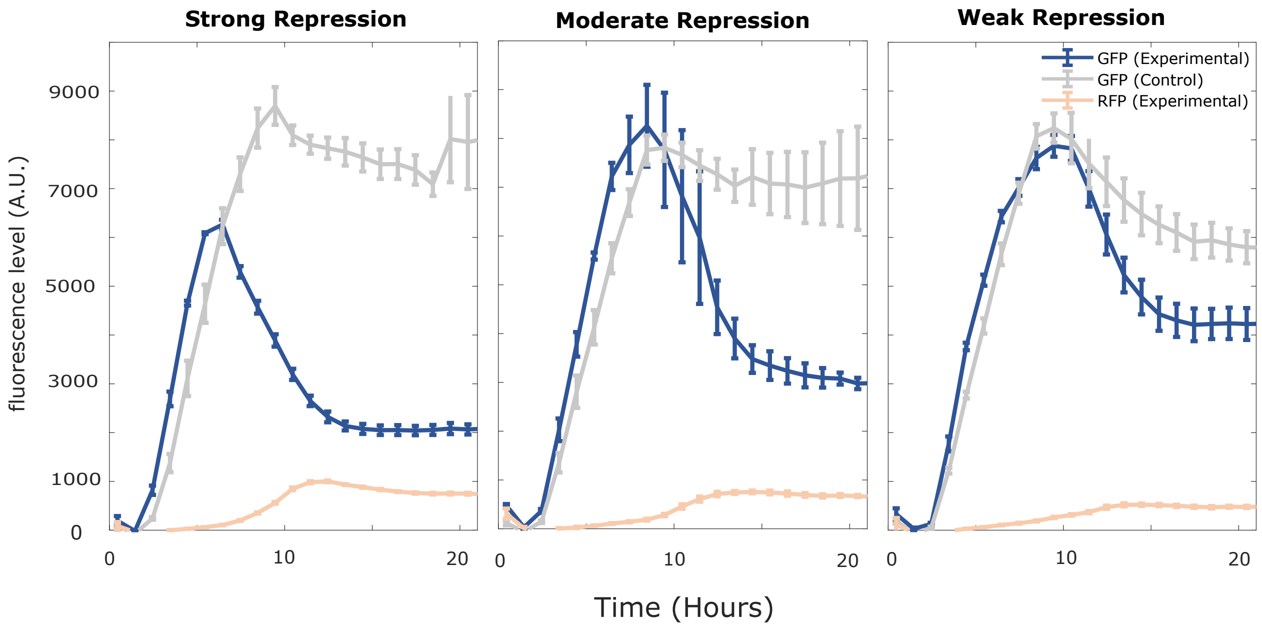

Figure S2. Non-normalized data showing in vivo growth response of experimental and control circuits over different repressive strengths shown in Figure 1I. (n = 4 biological replicates).

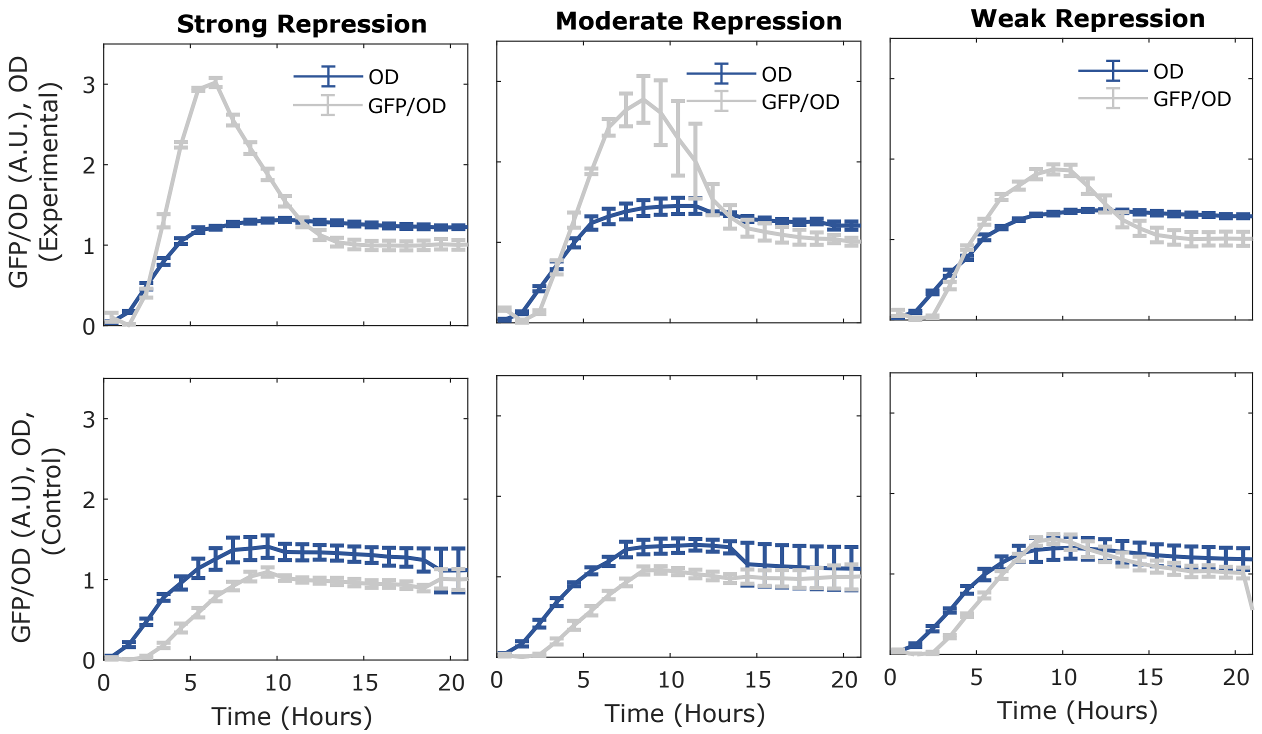

Figure S3. The growth (OD600) time-series data of experimental and control circuits over different repressive strengths are overlayed on the normalized GFP/OD data shown in Figure 1I. (n = 4 biological replicates).

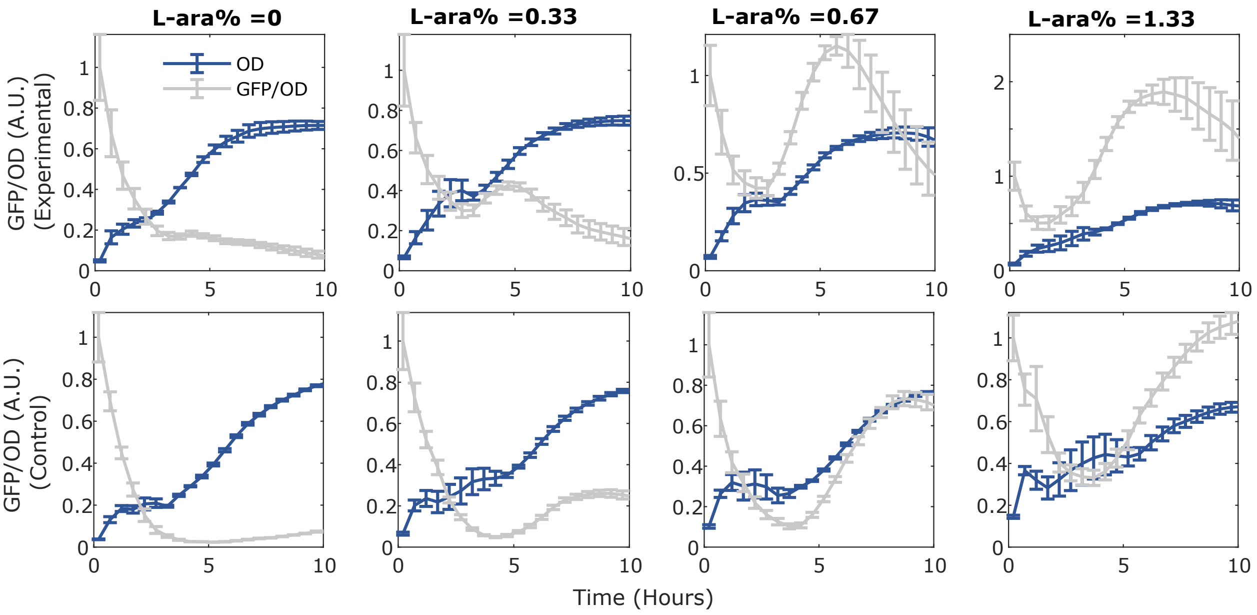

Figure S4. The growth (OD600) time-series data of the experimental and control circuits for four of eleven different L-ara concentrations are overlayed on the normalized GFP/OD data shown in Figure 2E. (n = 4 biological replicates).

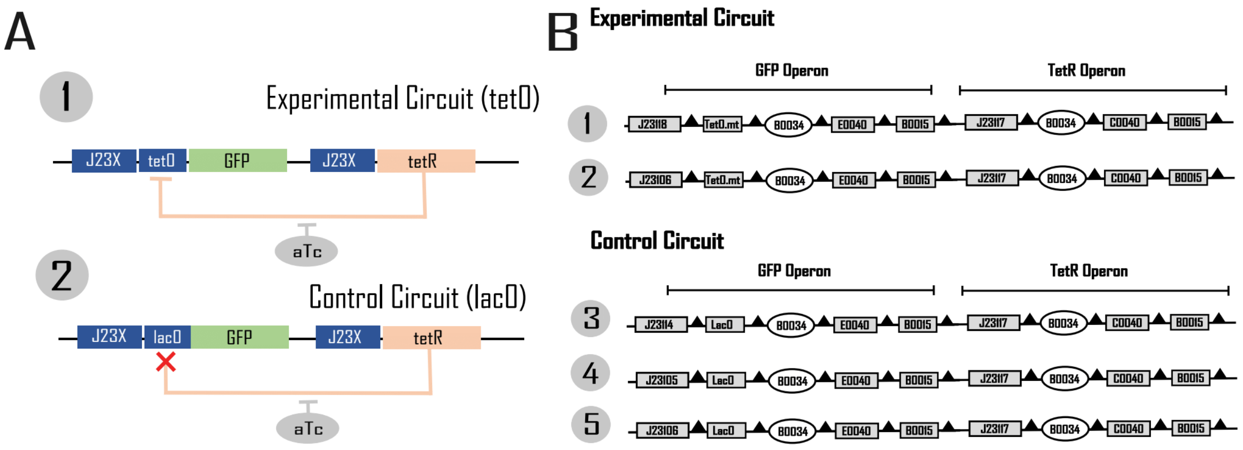

Figure S5. Design of the experimental and control simple circuits without a feedback loop. A) Schematic of the designed simple circuits without a feedback loop. In the experimental circuit, GFP as a general gene of interest is regulated by TetR , which binds to TetO regulatory sequence, while in the control circuit, it is not regulated by TetR as it does not bind to LacO regulatory sequence. B) The part specification for the circuit design with the sequence information in Table S2-3. Three control circuits (J23106-LacO, J23105-LacO, and J23114-LacO) and two experimental circuits (J23118-tetO and J23106-tetO) were created by placing GFP under constitutive promoters with either the LacO or TetO regulatory sequence alongside a constitutively expressed tetR module.

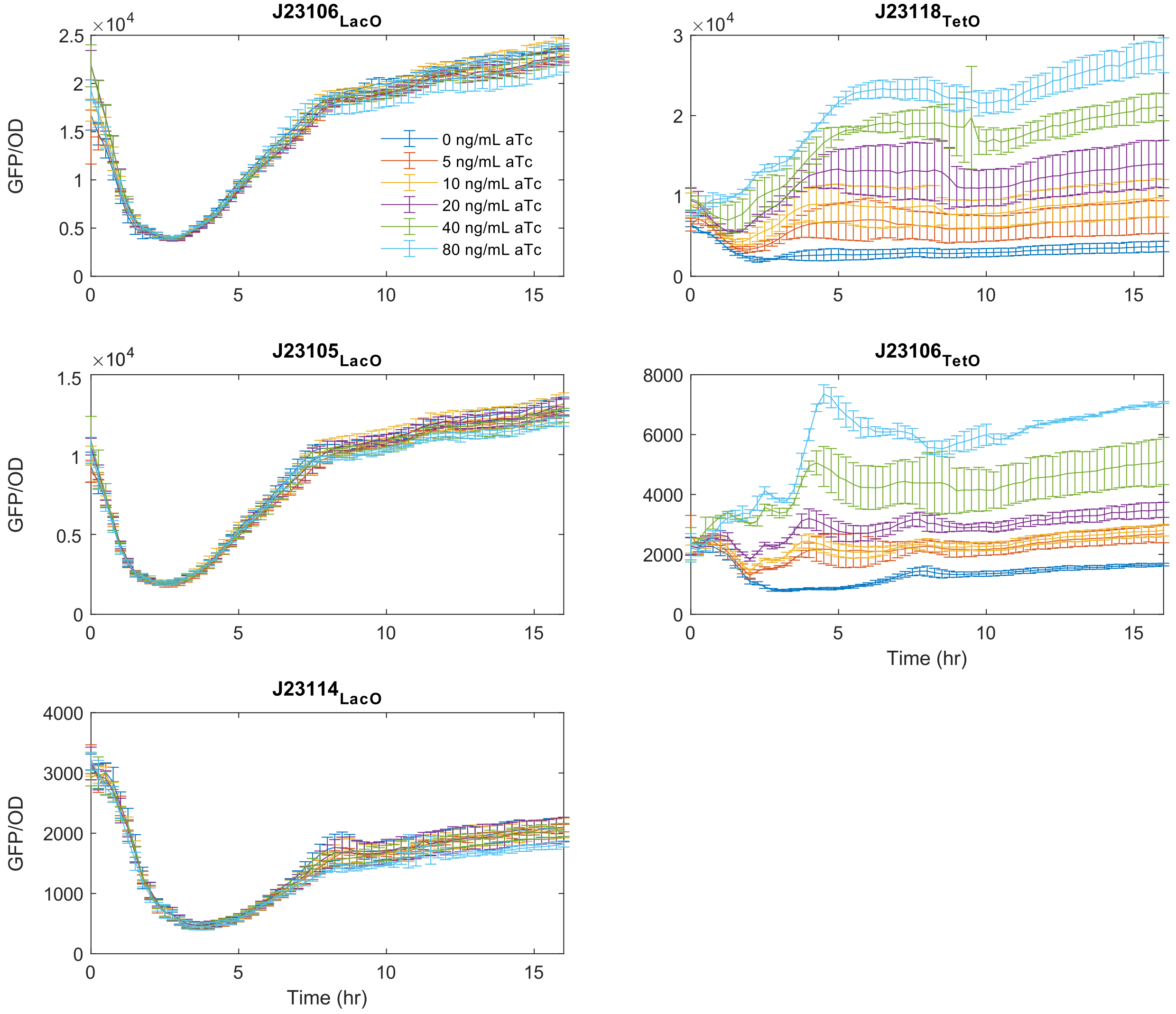

Figure S6. Time series data for simple GFP circuits under a range of aTc concentrations after dilution into fresh medium. While aTc has little to no effect on the control circuits (Left), the repression from TetR in the experimental circuits (Right) begins to attenuate as the aTc concentration rises, resulting in a rise in the GFP concentrations, aTc concentrations that result in matching activity between the experimental and control circuits were identified and utilized in the drop-rescue experiment in Figure S7. (n = 4 biological replicates).

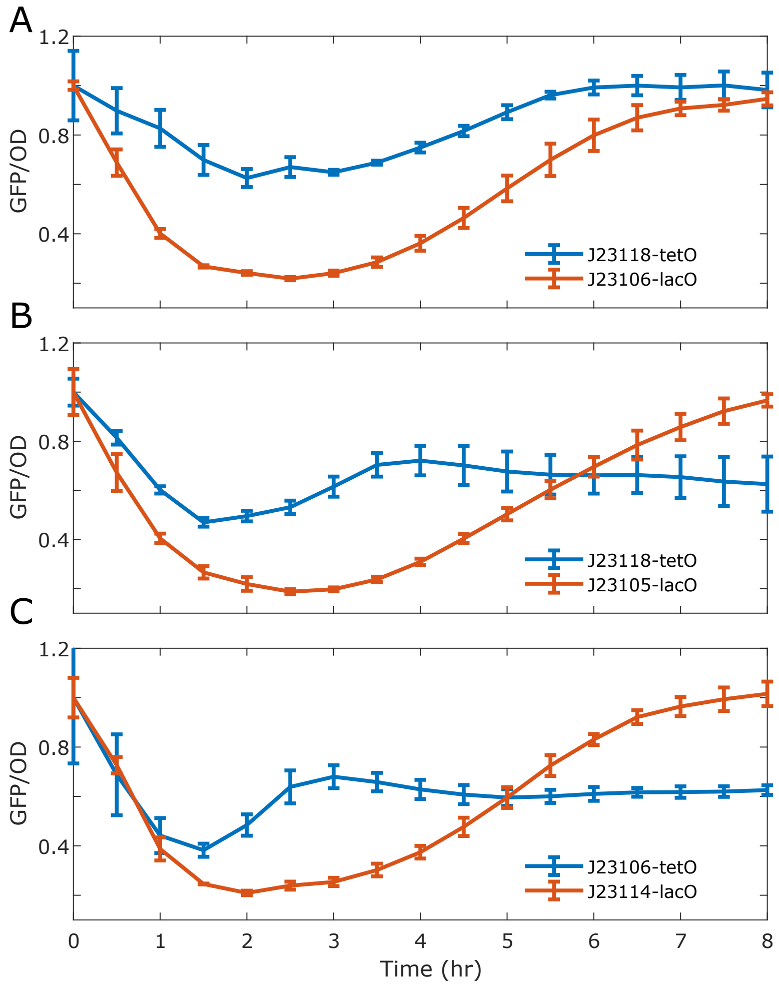

Figure S7. The GFP time-series data of three pairs of experimental and control circuits without a feedback loop. (A) experimental circuit with J23118-tetO promoter and control circuit with J23106-lacO promoter. 80 ng/ml aTc was used here. (B) experimental circuit with J23118-tetO promoter and control circuit with J23105-lacO promoter. 20 ng/ml aTc was used here. (C) experimental circuit with J23106-tetO promoter and control circuit with J23114-LacO promoter. 5 ng/ml aTc was used here. (n = 4 biological replicates).

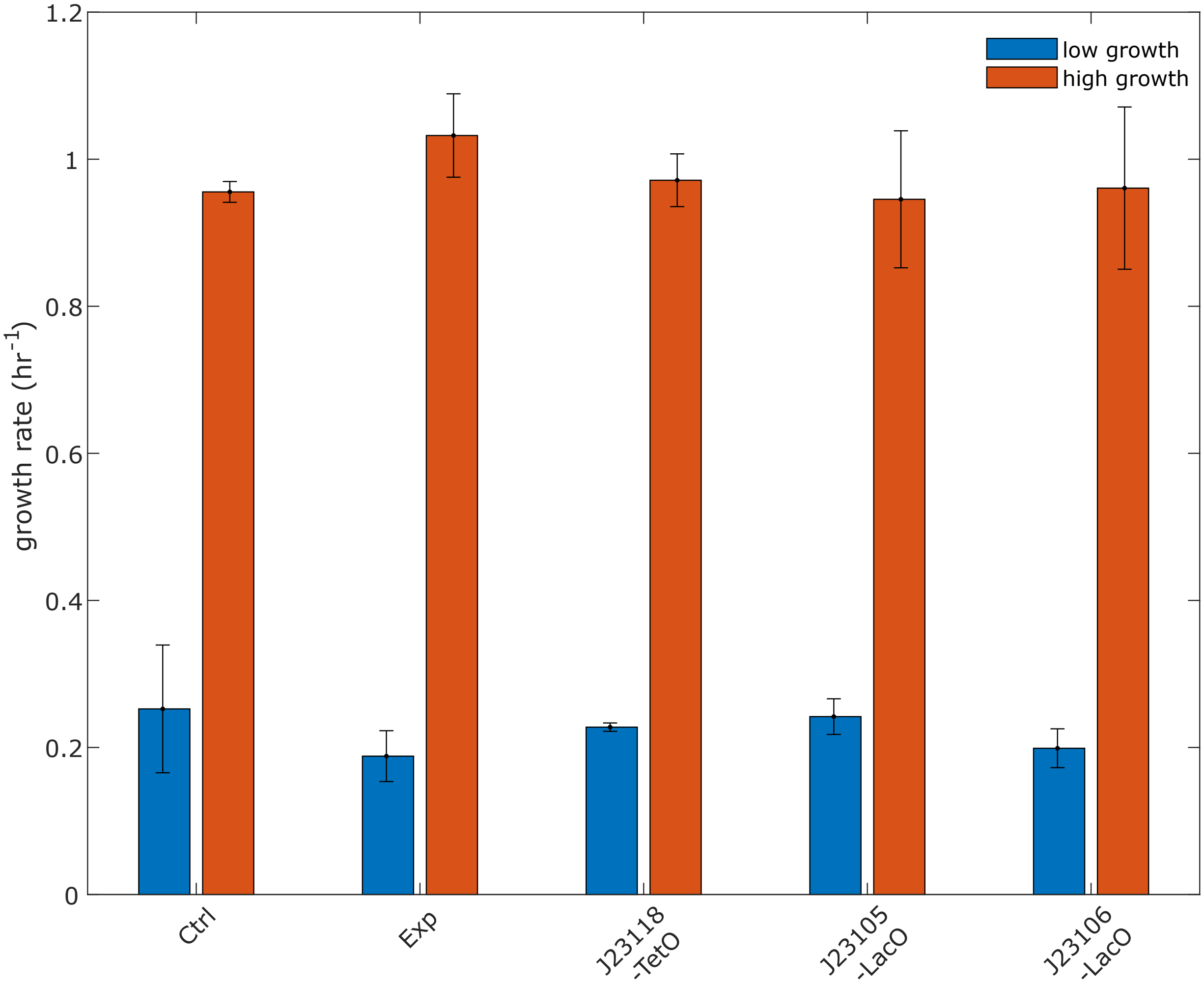

Figure S8. Growth rates between low and high growth conditions. (A) Growth rates for control and experimental circuits utilized in Figure 3E, and the simplified GFP circuits (J23118-TetO, J23105-LacO, J23106-LacO) utilized in Figure S6 under the low (28°C, 5% LB) and high growth condition (37°C, 100%LB). Data was collected at 15-minute intervals and fitted to an exponential regression model. (n = 4 biological replicates).

Table S1. Description and values of model parameters.

Parameter	Value	Description
k_g	0.9945	Growth constant
J_i	2.1919	Cellular burden factor
$k_{0,a}$	0.0335	Basal expression rate of AraC and GFP
$k_{1,a}$	0.6698	Active expression rate of AraC and GFP
$k_{0,t}$	0.25	Basal expression rate of TetR and RFP
λ	Variable	Hyperparameter for swapping between experimental and control scenarios (0 if control)
K_t	0.7000	Binding constant between TetR and its cognate promoter
K_{aTc}	0.4000	Binding constant between aTc and TetR
m	3	Hill coefficient for aTc-TetR binding
n_t	1.5	Hill coefficient for TetR binding to its cognate promoter
C_a^{min}	0.9 (0.63 for Figure 3A)	Minimum binding affinity between AraC and its cognate promoter
C_a^{max}	3 (2.1 for Figure 3A)	Maximum binding affinity between AraC and its cognate promoter
K_a	0.0019 (0.00285 for Figure 3A)	Michaelis-Menten constant for the binding between L-ara and AraC
d_a	0.3349	AraC/GFP Degradation Rate
d_t	0.1674	TetR/RFP Degradation Rate

Table S2. Sequence information for circuit design.

Symbol	Part Name	Sequence
▲	BB Scar	tgctatgcca
**K206000**	pBad Strong	acattgattatttgcacggcgtcacactttgctatgccatagcaagatagtccataagat<br>tagcggatcctacctgacgctttttatcgcaactctctactgtttctccat
**B0034**	Universal RBS	aaagaggagaaa
**C0080**	AraC	atggctgaagcgcaaaatgatcccctgctgccgggatactcgtttaacgcccatctg<br>gtggcgggtttaacgccgattgaggccaatggttatctcgatttttttatcgaccgaccg<br>ctgggaatgaaaggttatattctcaatctcaccattcgcggtcaggggtggtgaaaa<br>atcagggacgagaatttgtctgccgaccgggtgatattttgctgttcccgccaggaga<br>gattcatcactacggtcgtcatccggaggctcgcgaatggtatcaccagtgggtttact<br>ttcgtccgcgcgcctactggcatgaatggcttaactggccgtcaatatttgccaatacg<br>ggtttctttcgcccggatgaagcgcaccagccgcatttcagcgacctgtttgggcaaa<br>tcattaacgccgggcaaggggaagggcgctattcggagctgctggcgataaatctg<br>cttgagcaattgttactgcggcgcatggaagcgattaacgagtcgctccatccaccg<br>atggataatcgggtacgcgaggcttgtcagtacatcagcgatcacctggcagacag<br>caattttgatatcgccagcgtcgcacagcatgtttgcctgtcgccgtcgcgtctgtcaca<br>tcttttccgccagcagttagggattagcgtcttaagctggcgcgaggaccaacgcatc<br>agccaggcgaagctgcttttgagcactacccggatgcctatcgccaccgtcggtcgc<br>aatgttggttttgacgatcaactctatttctcgcgagtatttaaaaaatgcaccggggcc<br>agcccgagcgagttccgtgccggttgtgaagaaaaagtgaatgatgtagccgtcaa<br>gttgtcataataa
**B0015**	Universal Terminator	ccaggcatcaaataaaacgaaaggctcagtcgaaagactgggcctttcgttttatctg<br>ttgtttgtcggtgaacgctctctactagagtcacactggctcaccttcgggtgggcctttc<br>tgcgtttata
**E0040**	Green Fluorescent Protein	atgcgtaaaggagaagaacttttcactggagttgtcccaattcttgttgaattagatggt<br>gatgttaatgggcacaaattttctgtcagtggagagggtgaaggtgatgcaacatac<br>ggaaaacttacccttaaatttatttgcactactggaaaactacctgttccatggccaac<br>acttgtcactactttcggttatggtgttcaatgctttgcgagatacccagatcatatgaaa<br>cagcatgactttttcaagagtgccatgcccgaaggttatgtacaggaaagaactatat<br>ttttcaaagatgacgggaactacaagacacgtgctgaagtcaagtttgaaggtgata<br>cccttgttaatagaatcgagttaaaaggtattgattttaaagaagatggaaacattcttg<br>gacacaaattggaatacaactataactcacacaatgtatacatcatggcagacaaa<br>caaaagaatggaatcaaagttaacttcaaaattagacacaacattgaagatggaa<br>gcgttcaactagcagaccattatcaacaaaatactccaattggcgatggccctgtcct<br>tttaccagacaaccattacctgtccacacaatctgccctttcgaaagatcccaacgaa<br>aagagagaccacatggtccttcttgagtttgtaacagctgctgggattacacatggca<br>tggatgaactatacaaaaggcctgctgcaaacgacgaaaactacgctttagtagct
**C0040**	TetR	atgtccagattagataaaagtaaagtgattaacagcgcattagagctgcttaatgag<br>gtcggaatcgaaggtttaacaacccgtaaactcgcccagaagctaggtgtagagca<br>gcctacattgtattggcatgtaaaaaataagcgggctttgctcgacgccttagccattg<br>agatgttagataggcaccatactcacttttgccctttagaaggggaaagctggcaag<br>atttttacgtaataacgctaaaagttttagatgtgctttactaagtcatcgcgatggagc<br>aaaagtacatttaggtacacggcctacagaaaaacagtatgaaactctcgaaaatc<br>aattagcctttttatgccaacaaggtttttcactagagaatgcattatatgcactcagcgc<br>tgtggggcattttactttaggttgcgtattggaagatcaagagcatcaagtcgctaaag<br>aagaaagggaaacacctactactgatagtatgccgccattattacgacaagctatcg<br>aattatttgatcaccaaggtgcagagccagccttcttattcggccttgaattgatcatatg

		cggattagaaaaacaacttaaatgtgaaagtgggtccgctgcaaacgacgaaaac tacgctttagtagcttaataacactgatagtgctagtgtagatcac
**E1010**	Red Fluorescent Protein	atggcttcctccgaagacgttatcaaagagttcatgcgtttcaaagttcgtatggaagg ttccgttaacggtcacgagttcgaaatcgaaggtgaaggtgaaggtcgtccgtacga aggtacccagaccgctaaactgaaagttaccaaaggtggtccgctgccgttcgcttg ggacatcctgtccccgcagttccagtacggttccaaagcttacgttaaacacccggct gacatcccggactacctgaaactgtccttcccggaaggtttcaaatgggaacgtgtta tgaacttcgaagacggtggtgttgttaccgttacccaggactcctccctgcaagacgg tgagttcatctacaaagttaaactgcgtggtaccaacttcccgtccgacggtccggtta tgcagaaaaaaaccatgggttgggaagcttccaccgaacgtatgtacccggaaga cggtgctctgaaaggtgaaatcaaaatgcgtctgaaactgaaagacggtggtcact acgacgctgaagttaaaaccacctacatggctaaaaaaccggttcagctgccgggt gcttacaaaaccgacatcaaactggacatcacctcccacaacgaagactacacca tcgttgaacagtacgaacgtgctgaaggtcgtcactccaccggtgcttaataacgctg atagtgctagtgtagatcgc
**K206001**	pBad Weak	acattgattatttgcacggcgtcacactttgctatgccatagctttttatccataagattag cggatcctacctgacgctttttatcgcaactctctactgtttctccat
**LacO**	lacO Binding Sequence	aattgtgagcggataacaatttcacaca
**TetO**	Mutated tetO sequence*	tccctatcagtgatcga

Mutated tetO sequence was taken from [1].

Table S3. Constitutive promoters (Pc) used in the circuits.

Constitutive Promoters Used In Tuning (Pc)	Sequence
**J23103**	ctgatagctagctcagtcctagggattatgctagc
**J23109**	tttacagctagctcagtcctagggactgtgctagc
**J23114**	tttatggctagctcagtcctaggtacaatgctagc
**J23117**	ttgacagctagctcagtcctagggattgtgctagc
**J23105**	tttacggctagctcagtcctaggtactatgctagc
**J23106**	tttacggctagctcagtcctaggtatagtgctagc
**J23118**	ttgacggctagctcagtcctaggtattgtgctagc

References

[1] K. Lin *et al.*, “Mycobacterium tuberculosis Thioredoxin Reductase Is Essential for Thiol Redox Homeostasis but Plays a Minor Role in Antioxidant Defense,” *PLoS Pathog*, vol. 12, no. 6, p. e1005675, Jun. 2016, doi: 10.1371/JOURNAL.PPAT.1005675.

[2] R. Zhang *et al.*, “Topology-dependent interference of synthetic gene circuit function by growth feedback,” vol. 16, no. 6, pp. 695–701, doi: 10.1038/s41589-020-0509-x.
